# Enhancement of X-ray Induced Apoptosis by Mobile Phone-Like Radio-Frequency Electromagnetic Fields in Mouse Spermatocyte-Derived Cells

**DOI:** 10.3390/ijerph14060616

**Published:** 2017-06-07

**Authors:** Ke-Ying Zhang, Hui Xu, Le Du, Jun-Ling Xing, Bin Zhang, Qiang-Shan Bai, Yu-Qiao Xu, Yong-Chun Zhou, Jun-Ping Zhang, Yan Zhou, Gui-Rong Ding

**Affiliations:** 1 Department of Radiation Biology, Fourth Military Medical University, 169# ChangLe West Road, Xi’an 710032, China; zhangky@fmmu.edu.cn (K.-Y.Z.); cateyell@foxmail.com (L.D.); xingjunl@fmmu.edu.cn (J.-L.X.); xp10260641@163.com (J.-P.Z.); 2 Radiological College, Taishan Medical University, Taian 271000, China; hxu@tsmc.edu.cn; 3 Student Brigade, Fourth Military Medical University,169# ChangLe West Road, Xi’an 710032, China; flyinthesky_123@hotmail.com (B.Z.); leileiruirui@gmail.com (Q.-S.B.); 4 Department of Pathology, Fourth Military Medical University, 169# ChangLe West Road, Xi’an 710032, China; yuqiaoxu@fmmu.edu.cn; 5 Department of Radiation Oncology, Fourth Military Medical University, 169# ChangLe West Road, Xi’an 710032, China; zhouyongchun6@163.com

**Keywords:** RF field, X-ray, mouse spermatocyte-derived cells, proliferation, apoptosis

## Abstract

To explore the combined effects of environmental radio-frequency (RF) field and X-ray, mouse spermatocyte-derived (GC-1) cells were exposed to 1950 MHz RF field at specific absorption rate (SAR) of 3 W/kg for 24 h combined with or without X-ray irradiation at 6 Gy. After treatment, the cell proliferation level was determined by 3-(4,5-dimethyl-2-thiazolyl)-2,5-diphenyl-2-H-tetrazolium bromide (MTT) Assay and 5-Bromo-2-deoxy Uridine (BrdU) enzyme linked immunosorbent (ELISA) Assay. The apoptosis level was detected by annexin V flow cytometry assay, transferase-mediated deoxyuridine triphosphate-biotin nick end labeling (TUNEL) Assay and Caspase-3 Activity Assay. It was found that the proliferation and apoptosis level did not change in GC-1 cells after RF exposure alone. However, compared with the X-ray group, the proliferation level significantly decreased and the apoptotic rate significantly increased in the RF+X-ray group. Moreover, a significant decrease in Bcl-2 protein expression and increase in Bax protein expression were observed. The findings suggested that RF exposure at SAR of 3 W/kg did not affect apoptosis and proliferation in GC-1 cells by itself, but that it did enhance the effects of X-ray induced proliferation inhibition and apoptosis, in which B-cell lymphoma-2 (Bcl-2) and Bcl-2 associated X protein (Bax) might be involved.

## 1. Introduction

In recent years, the mobile phone has become one of the most indispensable communication tools. Accordingly, people are exposed to a complex environment of mobile phone-based radio-frequency (RF) field either with or without other radiation, for example X-ray. Besides possible carcinogenic effects, an association between mobile phone-based RF field and male reproduction has also been suggested because of the gradually increased incidence of infertility among men using mobile phones [1,2].

Some epidemiological studies suggest a possible link between the use of mobile phones and the decreased semen quality parameters [1,3], but the results are not consistent [4,5]. There is also some experimental evidence that exposures to RF field may lead to alteration of testes histology and disrupted spermatogenesis, but the results are also conflicting. An in vitro study reported that the RF field emitted by mobile phones decreased human sperm motility [6]. In addition, an obvious decrease in weight of the epididymis and seminal vesicles, seminiferous tubules diameter and tunica albuginea thickness, as well as an increase in head defects of sperm were reported after exposing rats to 2.4 GHz RF emitted from wireless fidelity (Wi-Fi) [7], which is consistent with an investigation by Tas et al. using a 900 MHz RF radiation emitted from a global system for mobile communication (GSM) signal generator [8]. Moreover, exposing male rats to 10 GHz microwave radiation showed a significant change in the level of reactive oxygen species (ROS), histone kinase, apoptotic cells, and percentage of G(2)/M transition phase of spermatozoa cell cycle [9]. In addition, RF field of 1800 MHz also increases in the generation of ROS, which may produce genotoxicity through oxidative DNA base damage in male germ cells [10]. However, investigations with no adverse effects were also reported. For instance, simultaneous exposure to code division multiple access (CDMA, 849 MHz) and wideband code division multiple access (WCDMA, 1.95 GHz) RF field at 4.0 W/kg specific absorption rate (SAR) did not lead to any observable adverse effects on the sperm count, testosterone concentration, malondialdehyde concentration, stages of spermatogenesis cyclein rats and appearance of apoptotic cells in testes [11]. Similarly, exposure for 8 weeks to simultaneous CDMA and WCDMA RF field did not affect the endocrine system since the serum levels of several hormones including sex hormone were not changed [12]. Moreover, the active (cleaved) caspase-3 levels, a well-known feature of typical apoptosis, in testes were not affected after exposing to a 900 MHz radiation 2 h/day (7 days/week) for 10 months [13].

Thus, the inconsistency of the RF effects on the reproduction system needs further studies. It was reported that the testis was one of the sensitive tissues to ionizing radiation [14] and RF field from mobile phones [2]. In modern society, people who are receiving radiotherapy, computer tomography (CT) are subjected to various kinds of ionizing radiations, and X-ray is a commonly used irradiation type. In addition, clinical radiologists might be subjected to X-ray. Meanwhile, these people are also sequentially or simultaneously exposed to an environmental RF field from mobile phone or base stations, or both. However, the combined effects of RF and X-ray have not been reported. Therefore, in this study, we first investigated the combined effects of a 1950 MHz RF field and X-ray on mouse spermatocyte-derived (GC-1) cells, which play a crucial role in spermatogenesis.

## 2. Materials and Methods

### 2.1. Cell Culture

BALB/c mouse spermatocyte-derived cell line (GC-1) was obtained from the Department of Pathophysiology of Fourth Military Medical University (original source-American Type Culture Collection, Manassas, VA, USA) and cultured at 37 °C in a 5% CO_2_ atmosphere. GC-1 cells were cultured in Dulbecco’s modified Eagle’s medium/F12 1:1 (DMEM/F121:1; Thermo Fisher Scientific, Waltham, MA, USA) with 10% fetal bovine serum (FBS) (HyClone, Logan, UT, USA) and 100 μg/mL penicillin streptomycin (Sigma-Aldrich, St. Louis, MO, USA). For all the experiments, 3 mL of a cell suspension was seeded into 35 mm Petri dishes (NuNc, Manassas, VA, USA) at a density of 1.5 × 10^4^ cells/mL at 24 h prior to RF exposure.

### 2.2. RF Field and X-ray Exposure

The RF exposure system was purchased from the Foundation for Information Technologies in Society (IT’IS Foundation, Zurich, Switzerland). The system consists of four parts: a RF generator, an arbitrary function generator, a narrow band amplifier and two rectangular waveguides. One waveguide is used for exposure, and the other waveguide is used for sham exposure. Both waveguides were placed in a CO_2_ incubator, the background direct current field and extremely low frequency electromagnetic field were shielded by the waveguides. The sensors and fans of the exposure system are connected to a computer that monitors the system during the exposure and maintains a constant temperature and environment for the waveguides (37 °C, 5% CO_2_/95% atmospheric air). The computer randomly selects one waveguide for exposure in each trial, and the temperature difference between the RF exposure and sham chambers does not exceed 0.1 °C.

At 24 h after cell seeding, the culture medium was replaced. Dishes were randomly divided into four groups: sham exposure group, RF group, X-ray group and RF+X group. Cells in the sham exposure group were placed in one rectangular waveguide which does not generate signal. RF group cells were placed in another rectangular waveguide which generates continuous Universal Mobile Telecommunications System (UMTS) signals at 1950 MHz, cells were exposed to RF field for 24 h, and the SAR was 3 W/kg. Based on previous report, cells in X-ray group were treated with X-ray at a dose rate of 4.268 Gy/min, and the total dose was 6 Gy, according to previous reports [15,16]. Cells in RF+X group were exposed to RF field for 24 h, and then treated with X-ray at a dose of 6 Gy. Cells in sham and RF groups were also transported to the X-ray facility and kept in the same condition but without X-ray transmission.

### 2.3. MTT Assay

GC-1 cell proliferation was measured by 3-(4,5-dimethyl-2-thiazolyl)-2,5-diphenyl-2-*H*-tetrazolium bromide (MTT) assay at 1d, 2d, 3d and 4d after RF and/or X-ray treatment. The cells were digested with 0.25% trypsin, and 1 × 10^3^ cells was seeded in 96 well plate in quintuplicate. Before detection, 20 μL MTT (5 mg/mL; Sigma-Aldrich) was added to each well and further incubated for 4 h. Cells were then solubilized in 150 μL dimethyl sulfoxide (Sigma-Aldrich). The absorbance was obtained using 96-well spectrophotometer (Bio-Rad, Hercules, CA, USA). The viability histograms were created by plotting the average of quintuplicate values calculated by optical measurements at 490 nm.

### 2.4. BrdU ELISA Assay

5-Bromo-2-deoxy Uridine (BrdU) has been proven to be a suitable marker for proliferating cells in numerous in vitro studies as well as in vivo studies. In this study, the level of cell proliferation in GC-1 cells at 3 d after RF and/or X-ray treatment were determined by BrdU enzyme linked immunosorbent (ELISA) assay kit (Calbiochem Merck, Whitehouse Station, NJ, USA). Briefly, 100 μL cells at 1 × 10^5^/mL were seeded per well into a 96-well culture dish, and allowed BrdU to label cells for 24 h in the incubator. After that, anti-BrdU antibody was added and incubated for 1 h at room temperature, then the reconstituted peroxidase goat anti-mouse immunoglobulin G (IgG) horseradish peroxidase (HRP) was added to the well. Finally, the absorbance of the reacted product was measured by using a spectrophotometric plate reader at dual wave lengths of 450–540 nm (Bio-Rad).

### 2.5. Flow Cytometry Analysis for Apoptosis

An annexin-V-fluorescein isothiocyanate (FITC) kit (BD Pharmingen, San Diego, CA, USA) was used to detect viable cells (annexin-FITC negative/propidium iodide (PI) negative), early apoptotic cells (annexin-FITC positive/PI negative), late apoptotic and necrotic cells (annexin-FITC positive/PI positive) and cells damaged during sample preparation (annexin-FITC negative/PI positive), according to the manufacture’ instructions. Fluorescence-activated cell sorter (FACS) analysis was performed on a FACS Calibur flow cytometry (BD Pharmingen, San Diego, CA, USA). GC-1 cells treated with 12 Gy of X-ray and collected at 3 d after irradiation was used as positive control.

### 2.6. TUNEL Assay

The apoptosis level of GC-1 cells after RF and/or X-ray treatment was evaluated by the transferase-mediated deoxyuridine triphosphate-biotin nick end labeling (TUNEL) kit (Roche, Basel, Switzerland) according to the manufacturer’ instructions. Briefly, GC-1 cells were fixed in 4% paraformaldehyde in PBS (pH 7.4) for 10 min at 4 °C, after washing with PBS, permeabilization solution (0.1% Triton X-100 in 0.1% sodium citrate) was added to the cells for 2 min on ice. The TUNEL reaction mixture (enzyme solution and label solution) was added and the slides were incubated for 60 min at 37 °C in a humidified atmosphere in the dark. After that, the cells were washed with PBS, and then, nuclear counterstaining was performed with 4′,6-diamidino-2-phenylindole dihydrochloride (DAPI, 0.1μg/mL; Sigma-Aldrich). Finally, the cells were observed and analyzed in a “blinded” fashion under a fluorescence microscope (Leica Microsystems, Heidelberg, Germany). TUNEL-positive cells showed green fluorescence. At least a minimum of 500 nuclei from 8 random fields were counted. The results were expressed as percentage of TUNEL-positive cells.

### 2.7. Caspase-3 Activity Assay

The activity of Caspase-3 in GC-1 cells after RF and/or X-ray treatment was measured by a Caspase-3 activity assay kit (Beyotime Institute of Biotechnology, Shanghai, China). The cells were digested with 0.25% trypsin and then mixed with the cell lysis buffer. After incubating on ice for 30 min, the cell lysis mixture was centrifuged at 10,000 g at 4 °C. The supernatant was used for caspase-3 activity assay according to manufacturer’s instructions. Colorimetric reaction was measured at 405 nm in a microtiter plate reader (Bio-Rad).

### 2.8. Western Blot Analysis and Antibodies

After treatment, cells were washed twice with cold PBS and then pelleted, and then cell lysis reagent (Sigma-Aldrich) was added to the cell pellet for protein extraction. Protein concentration was determined by bovine serum albumin (BSA) assay using BSA as the reference, and the absorbance of the reacted product was measured at wavelength of 540–595 nm. Samples were boiled for 3 min before loading onto 12% sodium dodecyl sulfate (SDS)-polyacrylamide gel. After electrophoresis, the gels were electro blotted onto a polyvinylidene fluoride (PVDF) membranes (Merck Millipore, Whitehouse Station, NJ, USA). The antibodies used were as follows: anti-Bcl-2 rabbit polyclonal antibody (Abcam, Cambridge, MA, USA), anti-Bax rabbit polyclonal antibody (Abcam), anti-β-actin rabbit polyclonal antibody (Santa Cruz Biotechnology Inc., Dallas, TX, USA), and anti-rabbit IgG HRP (Abcam). The blot was visualized with an enhanced chemiluminescence (ECL) kit (Merck Millipore) and exposed to ECL Hyperfilm.

### 2.9. Statistical Analysis

All experimental data were expressed as mean ± standard deviation (SD). MTT, BrdU and Western blots data was examined by ANOVA followed by Dunnett-test, and the residuals were verified to have a near normal distribution. Flow Cytometry Analysis for Apoptosis data was examined by non-parametric statistical test. All experiments were repeated at least three times on independent samples. Data analysis was performed using SPSS 17.0 software (SPSS Inc., Chicago, IL, USA). Values of *p* less than 0.05 were considered statistically significant.

## 3. Results

**Figure 1:**
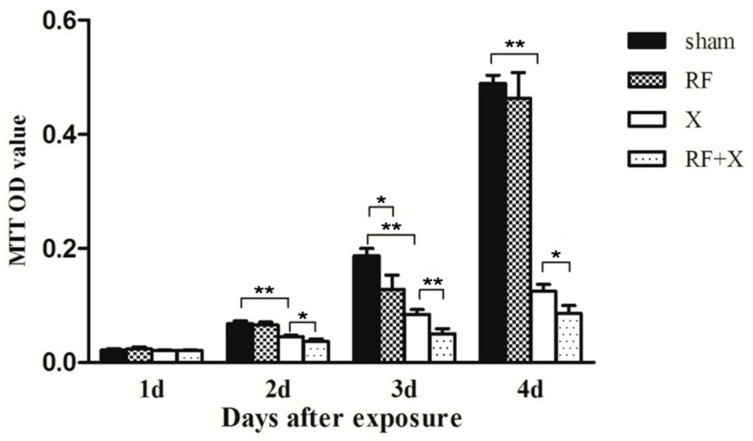
Cell proliferation level of GC-1 cells at different time points after treatment with radio-frequency (RF) and/or X-ray detected by MTT assay. Bars represent the means ± standard deviation (SD). * *p* < 0.05; ** *p* < 0.01; *n* ≥ 3; X: X-ray; MTT: 3-(4,5-dimethyl-2-thiazolyl)-2,5-diphenyl-2-H-tetrazolium bromide.

**Figure 2:**
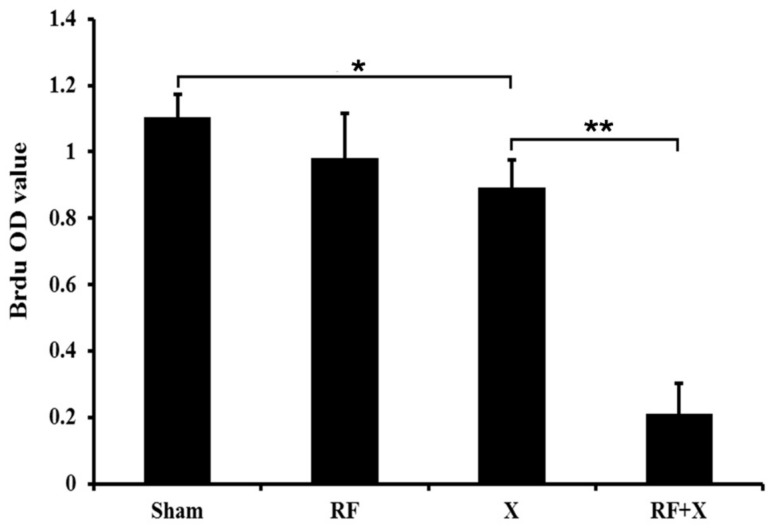
Cell proliferation level of GC-1 cells at 3d after treatment with RF and/or X-ray detected by bromodeoxyuridine (BrdU) ELISA assay. Bars represent the means ± SD. * *p* < 0.05; ** *p* < 0.01; *n* ≥ 3; ELISA: enzyme-linked immunosorbent assay.

**Figure 3:**
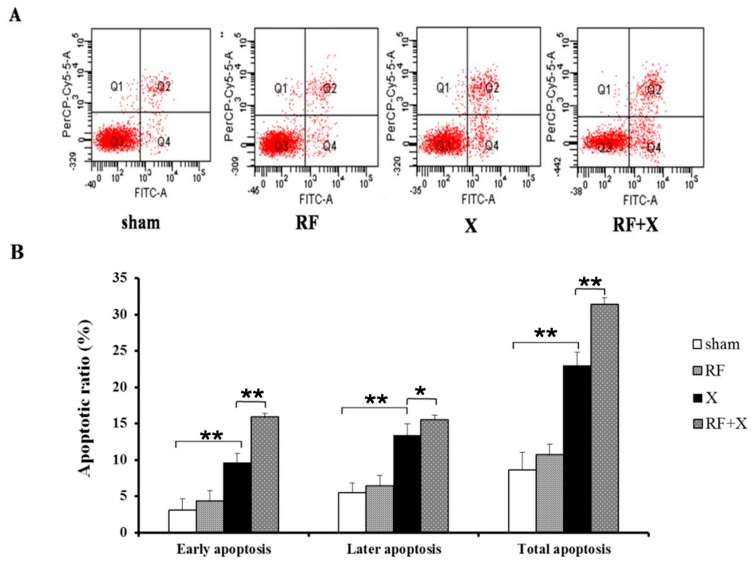
The apoptotic ratio in GC-1 cells at 3d after treatment with RF and/or X-ray, measured by Annexin V flow cytometry; * *p* < 0.05, ** *p* < 0.01; *n* ≥ 3; FITC-A: fluorescein isothiocyanate-A; PerCP-Cy5-5-A: peridinin chlorophyll protein-cyanine 5-5-A.

**Figure 4:**
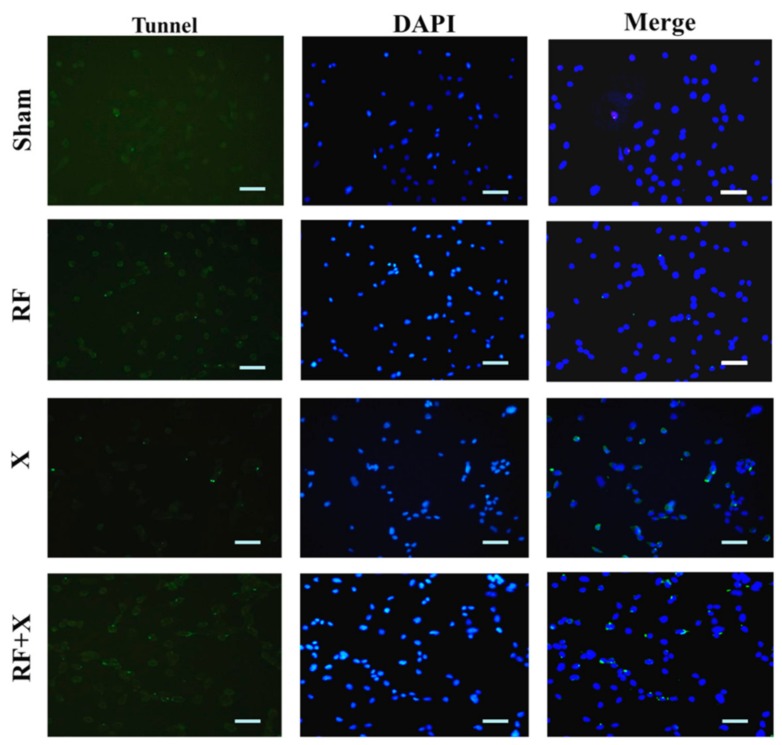
The apoptosis level in GC-1 cells at 3d after treatment with RF and/or X-ray detected by transferase-mediated deoxyuridine triphosphate-biotin nick end labeling (TUNEL) staining. TUNEL staining (green) indicates apoptotic nuclei, 4’,6-diamidino-2-phenylindole (DAPI) counterstaining (blue) indicates nuclei (Scale bar = 50 μm); Representative images were chosen.

**Figure 5:**
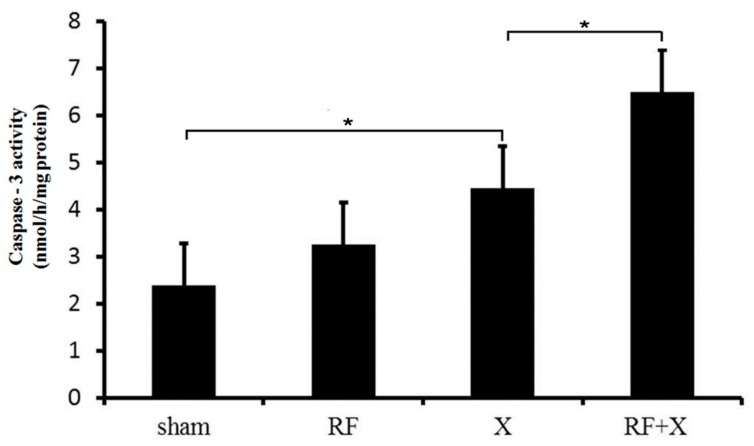
The activity of Caspase-3 in mouse spermatocyte-derived (GC-1) cells after treatment with RF and/or X-ray. *p < 0.05; *n* ≥ 3.

**Figure 6:**
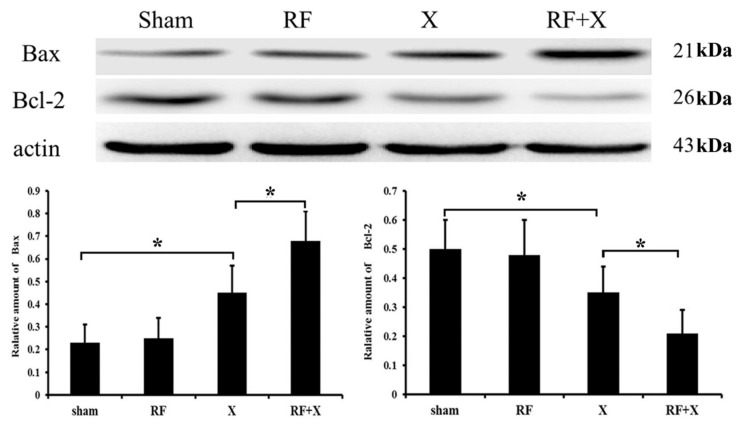
The protein level of apoptosis related genes in GC-1 cells determined by western blot after treatment with RF and/or X-ray. * *p* < 0.05; *n* ≥ 3.

The data presented in Figure 1, Figure 2, Figure 3, Figure 4, Figure 5 and Figure 6 were mean ± SD from at least 3 independent experiments. The statistical differences between RF and sham, between X-ray and sham, between X-ray and RF+X-ray are represented in the Figures.

### 3.1. Cell Proliferation of GC-1 Cells after Exposure to RF and/or X-ray Determined by MTT Assay

MTT assay showed that there was no difference in cell proliferation level between sham group and RF group at 1d, 2d and 4d after treatment, on day 3, the cell proliferation level in RF group slightly decreased(*p* < 0.05). The cell proliferation level was significantly reduced in the X-ray group at 2d, 3d and 4d after X-ray treatment (*p* < 0.01) compared with sham group. Moreover, RF exposure aggravated X-ray-induced cell proliferation inhibition (Figure 1).

To confirm the results of MTT assay, BrdU ELISA assay was performed with cells from the same exposure session. It was shown that there was no difference in cell proliferation level of GC-1 cells between the sham group and RF group. However, the cell proliferation level was significantly reduced in the X-ray group compared with the sham group at 3d after X-ray treatment (*p* < 0.05). Moreover, RF exposure aggravated X-ray-induced cell proliferation inhibition which is consistent with the results of MTT assay (Figure 2).

### 3.2. The Apoptosis Level of GC-1 Cells after Exposure to RF and/or X-ray

To investigate the effects of RF exposure combined with X-ray on apoptosis in GC-1 cells, annexin V flow cytometry assay was performed. Immediately after 24hof exposure to 1950 MHz RF field and/or X-ray treatment, the number of early apoptotic cells, later apoptotic cells and total apoptotic cells did not exhibit any obvious changes in comparison with sham exposed cells. However, at 3d after exposure to X-ray, the number of apoptotic cells significantly increased compared with sham group, and exposure to a RF field for24 h significantly increased the apoptotic rate induced by X-ray. And at 3d after only exposure to RF field for 24 h, the number of apoptotic cells increased lightly, but did not show significantly changes compared with sham group (Figure 3).

To confirm the results of flow cytometry, TUNEL assay was performed at 3 d after exposure to RF and/or X-ray. As shown in Figure 4, few apoptotic cells were found in the sham (3.1% ± 0.5%) and RF group (3.5% ± 0.4%). However, after X-ray irradiation, the number of apoptotic cells obviously increased (13.7% ± 1%). Moreover, RF exposure promoted X-ray-induced cell apoptosis level (20.1% + 1.5%) (Figure 4).

### 3.3. The Activity of Caspase-3 in GC-1 Cells after Treatment with RF and/or X-ray

As shown in Figure 5, there was no difference in caspase-3 activity level of GC-1 cells between sham group and RF group. At 24 h after X-ray irradiation, the activity of caspase-3 increased significantly compared with the sham group (*p* < 0.05). Moreover, RF exposure promoted X-ray-induced caspase-3 activation, which was consistent with the results of flow cytometry and TUNEL assay.

### 3.4. Protein Expression after Treatment with RF and/or X-ray

To explore the mechanisms by which exposure to RF promote X-ray-induced apoptosis, the expression level of apoptosis-related proteins was examined. As shown in Figure 6, no differences in Bcl-2 and Bax expression were demonstrable between RF group and sham group. In addition, the protein expression of Bcl-2 significantly decreased and Bax significantly increased in X-ray group. Moreover, RF exposure aggravated X-ray-induced Bcl-2 expression inhibition and Bax expression promotion.

## 4. Discussion

Spermatogenesis in testes begins with spermatogonia, which is essential for normal reproductive function. Moreover, spermatogenesis is susceptive to environmental risk factors, such as irradiation [14] and RF filed [2]. It was found that the total number of spermatogonia obviously declined after treatment with 5 Gy of irradiation [17]. The block for spermatogenesis progression and the induced abnormal sperm after RF exposure were also reported [7,8,18]. Recent study showed that the use of mobile phone hands-free devices lowered the RF exposure to the brain. Accordingly, it might increase the RF exposure to the gonads [19]. In addition, as we know, under some conditions, people might be exposed to both the irradiation and RF field, such as clinical radiologists as well as the patients who are receiving X-ray radiotherapy, CT or X-ray examination who might encounter X-rays and RF field emitted from mobile phones. However, the combined effects of RF and X-ray on male reproductive health have not been reported. In the present study, we performed experiments to detect the possible impact of RF exposure either with or without X-ray on spermatogonia. Since MTT assay and BrdU incorporation were widely used to determine cell proliferation [20], we firstly used these two methods to examine cell proliferation in GC-1 cells after RF exposure with or without X-ray irradiation. It was found that although RF exposure did not affect the proliferation level in GC-1 cells, it could aggravate X-ray-induced cell proliferation inhibition, which has not been reported before. Although slight cell proliferation inhibition was observed in the RF group at day 3 after treatment detected by MTT assay, BrdU incorporation assay failed to confirm this result. Considering all the data, we do not think that RF exposure alone could obviously affect cell proliferation in GC-1 cells.

Our next investigation showed that apoptosis was not induced in GC-1 cells after exposure to RF alone, which was consistent with the report by Dasdag et al. that 2 h/day (7 days/week) exposure of 900 MHz RF field over a period of 10 months did not induce apoptosis in rat testes [13]. However, the RF exposure aggravated X-ray-induced apoptosis in GC-1 cells, and this was supported by three evidences. First, annexin V flow cytometry analysis showed that the percent of apoptotic cells increased significantly in the RF+X-ray group, compared with the X-ray group. The second evidence came from TUNEL assay, in which much more DNA fragmentations associated with apoptotic cell death in RF+X group were detected than that in X-ray group. Similarly, after irradiation of 10 Gy as a single dose to Wistar albino male rats, the TUNEL-positive cells were frequently detected in spermatogonia [21]. Finally, the increased activity for caspase-3 also supported that RF exposure promoted X-ray-induced apoptosis in GC-1 cells.

It has been well documented that the genes encoding Bcl-2 and Bax are involved in the process of apoptosis which is initiated by a death inducing stimulus [22,23,24,25]. Cells showing a higher expression of Bax undergo apoptosis, while those overexpressing Bcl-2 often undergo suppression of apoptosis [26]. In our study, the protein expression of Bcl-2 significantly decreased and Bax significantly increased in the X-ray group and RF exposure aggravated X-ray-induced Bcl-2 expression inhibition and Bax expression promotion, which was consistent with the results of apoptosis. These findings indicate that Bcl-2 and Bax might be involved in the mechanism by which RF exposure aggravates X-ray-induced apoptosis in GC-1 cells.

Although the combined effects of RF and X-ray on male reproductive health have not been reported, some researchers have observed the adaptive response of RF in some cell lines. It was found that pre-exposure to an RF field protected cultured cells from the damaging effects of ionizing radiation [27,28]. For example, human blood lymphocytes exposed to 1950MHz RF fields resulted in a resistance to subsequent 1.0 or 1.5 Gy X-irradiation induced DNA damage. However, in this study, we failed to establish the adaptive effects of 1950MHz RF exposure on 6 Gy X-ray-induced cell proliferation inhibition and apoptosis in GC-1 cells. The inconsistency of the results might be due to the different cell types, intensities of RF field and/or X-ray, detected endpoints, etc., in different studies.

Until now, various parameters of RF exposure were used to investigate its biological effects, and SAR was one of the important parameters of the RF field. In the present study, SAR 3.0 W/kg was selected based on the 2.0 W/kg limit by the International Commission on Nonionizing Radiation Protection (ICNIRP) and Institute of Electrical and Electronics Engineers (IEEE) [29,30]. After 24 h SAR 3.0 W/kg RF exposure, the temperature in culture medium increased by less than 0.1 °C, which indicated that no thermal effect was involved in the proliferation inhibition and apoptosis induced by RF and X-ray treatment. In the present study, only one SAR value (3.0 W/kg) was investigated instead of a range of SAR values. Whether the enhancement of X-ray induced apoptosis by RF exposure has the intensity dependence was unknown. Moreover, what is the threshold value of RF field? To answer these questions, further studies are needed.

## 5. Conclusions

Although exposure to a RF field alone cannot affect cell proliferation and apoptosis in GC-1 cells, it could aggravate cell proliferation inhibition and apoptosis induced by X-ray, in which Bcl-2 and Bax might be involved.
